# Depression in patients with knee osteoarthritis: risk factors and associations with joint symptoms

**DOI:** 10.1186/s12891-020-03875-1

**Published:** 2021-01-07

**Authors:** Shuang Zheng, Liudan Tu, Flavia Cicuttini, Zhaohua Zhu, Weiyu Han, Benny Antony, Anita E. Wluka, Tania Winzenberg, Dawn Aitken, Leigh Blizzard, Graeme Jones, Changhai Ding

**Affiliations:** 1 Department of Rheumatology, The First Affiliated Hospital of Anhui Medical University, Hefei, Anhui China; 2 Menzies Institute for Medical Research, University of Tasmania, Private Bag 23, Hobart, Tasmania Australia; 3 Department of Rheumatology, The Third Affiliated Hospital of SUN YAT-SEN University, Guangzhou, China; 4 Department of Epidemiology and Preventive Medicine, Monash University, Melbourne, Victoria Australia; 5 Clinical Research Centre, Zhujiang Hospital, Southern Medical University, Guangzhou, Guangdong China

**Keywords:** Depression, Knee osteoarthritis, Risk factors, Joint symptoms

## Abstract

**Background:**

To describe demographic and clinical factors associated with the presence and incidence of depression and explore the temporal relationship between depression and joint symptoms in patients with symptomatic knee osteoarthritis (OA).

**Methods:**

Three hundred ninety-seven participants were selected from a randomized controlled trial in people with symptomatic knee OA and vitamin D deficiency (age 63.3 ± 7.1 year, 48.6% female). Depression severity and knee joint symptoms were assessed using the patient health questionnaire (PHQ-9) and the Western Ontario and McMaster Universities Osteoarthritis Index (WOMAC), respectively, at baseline and 24 months.

**Results:**

The presence and incidence of depression was 25.4 and 11.2%, respectively. At baseline, having younger age, a higher body mass index (BMI), greater scores of WOMAC pain (PR: 1.05, 95%CI:1.03, 1.07), dysfunction (PR: 1.02, 95%CI:1.01, 1.02) and stiffness (PR: 1.05, 95%CI: 1.02, 1.09), lower education level, having more than one comorbidity and having two or more painful body sites were significantly associated with a higher presence of depression. Over 24 months, being female, having a higher WOMAC pain (RR: 1.05, 95%CI: 1.02, 1.09) and dysfunction score (RR: 1.02, 95%CI: 1.01, 1.03) at baseline and having two or more painful sites were significantly associated with a higher incidence of depression. In contrast, baseline depression was not associated with changes in knee joint symptoms over 24 months.

**Conclusion:**

Knee OA risk factors and joint symptoms, along with co-existing multi-site pain are associated with the presence and development of depression. This suggests that managing common OA risk factors and joint symptoms may be important for prevention and treatment depression in patients with knee OA.

**Trial registration:**

ClinicalTrials.gov identifier: NCT01176344.

Anzctr.org.au identifier: ACTRN12610000495022.

**Supplementary Information:**

The online version contains supplementary material available at 10.1186/s12891-020-03875-1.

## Background

Osteoarthritis (OA) is a prevalent joint disease, characterised by whole joint structural changes, and is considered as a serious disease [1]. OA affects nearly 240 million people throughout the world, and its prevalence is projected to increase as the population ages and obesity rates increase [2]. Joint pain, stiffness and limited function are common joint symptoms of OA, resulting in reduced quality of life and disability that contribute to substantial financial burden for individuals [3]. In addition, OA is associated with comorbidities, including cardiovascular disease, diabetes, hypertension, falls, fractures and depression [1].

Depression is a major global public-health issue and is projected to be the second leading cause of disease burden by the year 2020 [4, 5]. A recently systematic review and meta-analysis recently reported that 19.9% of people with OA had depressive symptoms, with a relative risk of depression of 1.17 in those with OA compared to those without [6, 7]. However, depression is often under-recognised and under-treated in older adults, particulary in patients with OA [8, 9]. Furthermore, concomitant depression in OA patients contributes to increased difficulties in OA management and disease burden [4]. Therefore, the interaction between OA and depression should be taken seriously, and screening, prevention and treatment of depression in OA patients should be considered [10].

Concomitant depression with OA may be mediated by either biological or behavioural mechanisms with different aetiology and risk factors [4, 11]. For example, there may be a mechanism between pain and depression by neurobiological links [12]. Besides, physical limitation caused by OA may lead to gradual withdrawal from rewarding activities and social activities [13]. A better understanding of depression in OA patients is crucial for identifying modifiable risk factors and key areas for intervention [1]. Joint pain, decreased physical performance and increased risks for chronic comorbidities are typical characteristics of OA, which may be associated with depression [9, 14, 15]; however, the longitudinal relationship between clinical OA characteristics and depression in individuals with OA has been poorly studied. In addition, although the reciprocal relationship between depression severity and pain is established, whether current depression severity predicts changes in joint symptoms overtime has been rarely investigated in knee OA patients [16–18].

The aims of this study were, therefore, to describe demographic and clinical factors associated with the presence and incidence of depression and to explore the temporal relationship between depression and joint symptoms in patients with symptomatic knee OA.

## Methods

### Study design and participants

This study is a post-hoc analysis of a multicenter, randomized, double-blind, placebo-controlled trial, the Vitamin D Effect on Osteoarthritis (VIDEO) study, aimed to evaluate the effect of vitamin D supplementation in patients with symptomatic knee OA and vitamin D deficiency [19]. Participants were allocated to either the treatment or placebo arm at a ratio of 1:1, and received a capsule containing 50,000 IU (1.25 mg) vitamin D_3_ (cholecalciferol) or placebo monthly for 24 months [19]. In this current study, both treatment and placebo groups were combined together as a cohort, which adheres to STROBE statement and include a completed STROBE checklist in the supplementary file.

Participants who suffered from symptomatic knee OA (American College of Rheumatology criteria) for at least 6 months in Tasmania and Victoria (Australia) were enrolled [19, 20]. Exclusion criteria were detailedly described in the protocol [19]. The important exclusion criteria were participants with the severe radiographic changes (grade 3 on the Altman and Gold atlas) and/or severe knee pain on standing (> 80 mm on a 100-mm VAS) [19].

### Anthropometrics and social demographic characteristics

Height and weight were measured to the nearest 0.1 cm and 0.1 kg (with shoes and bulky clothing removed) using electronic scales (Heine S-7307, Heine, New Hampshire, USA) and stadiometer (Leicester Height Measure, Invicta Plastics Ltd., Leicester, UK) at baseline. Body mass index (BMI, in kg/m^2^) was calculated [19]. The participants’ information of education history, work status/types, current smoking and concomitant medication usage were collected by the questionnaire at baseline.

### Outcomes measurements

#### Depression severity

Depression severity was measured using the patient health questionnaire (PHQ-9) at baseline and 24 month. PHQ-9 is a validly, reliably and commonly used instrument in diagnosing and assessing the severity of depression [21]. It contains nine items with a score range of 0 to 27, with each item being scored from 0 (not at all) to 3 (nearly every day). In this current study, the cut-off point of ≥5 for PHQ-9 score at baseline was defined as prevalent depression (no depression or depression), and PHQ-9 score of ≥5 at follow-up in those without depression (PHQ-9 score < 5) was defined as incident depression. Besides, based on the generally recommended criterion, a cut-off point of ≥5 has a sensitivity of 81.5% and a specificity of 80.6% for mild depression and a cut-off point of ≥10 has a sensitivity of 54.3% and a specificity of 91.1% for moderate or severe depression [22]. The severity of depression (none, mild and moderate, severe) was used to explore the association between baseline depressive severity and change in knee symptoms over 24 months.

### Predictors measurement

#### Knee joint symptoms

The Western Ontario and McMaster Universities OA Index (WOMAC) was used to evaluate knee joint symptoms at baseline and 24 month. The index is widely used to assess the joint functional capacity in clinical trials [23]. Three subscales (pain, stiffness, and physical function) and 24 questions (5 related to pain, 2 to stiffness and 17 to physical function) constitutes the index, with scores from 0 (none) to 100 (severe). The sum of three subscales scores was calculated as the total WOMAC score (0–2400). The WOMAC score was used in the analyses as a continuous variable.

#### Multi-site pain

Participants were asked whether they experienced (yes/no) neck, lower back, hands, shoulders and others pain at baseline. The total number of painful locations (range 0 to 5) was categorized into three groups (no pain, one painful site, two or more painful sites).

#### Self-reported medical conditions

Participants were asked whether they have been diagnosed (yes/no) by a doctor or a nurse with any of the following conditions: depression, angina, high blood pressure, heart attack, stroke, high cholesterol, diabetes, osteoporosis, asthma, bronchitis and emphysema, and whether they had these conditions currently. The total number of current comorbidities, except for depression, was categorized into three groups (no comorbidity, one comorbidity and two or more comorbidities).

### Data analyses

Baseline characteristics are described as mean ± standard deviation (SD) or numbers of participants (percentage). Univariable and multivariable log-binomial regressions were used to explore risk factors associated with the presence of depression at baseline and incidence of depression over time. If the log binomial model failed to converge, it was estimated by using a poisson distribution and robust standard errors. Multivariable models for prevalent depression were adjusted for age, sex, BMI and baseline vitamin D level. Multivariable models for incident depression were adjusted for age, sex, BMI, baseline 25-hydroxyvitamin D level and treatment arms (vitamin D treatment versus placebo). Univariable and multivariable linear regressions were used to examine the temporal relationship between baseline depressive severity and change in knee symptoms over 24 months before and after adjustment for age, sex, BMI, baseline 25-hydroxyvitamin D level, treatment arms and baseline WOMAC score. All tests were two-sided and a *P* value of < 0.05 was considered as statistically significant. Stata version 12.0 was used to perform statistical analyses.

## Results

### Baseline characteristics of participants

Table 1 presents baseline characteristics of the study participants. The mean age of 397 participants was 63.3 ± 7.1 years and mean BMI was 29.6 ± 4.9. Of them, 196 (49.4%) participants were female and 201 (50.6%) were allocated to the treatment group. At baseline, 296 (74.6%) participants were identified as not suffering from depression, 70 (17.6%) were identified as suffering from mild depression, and 31 (7.8%) were identified as suffering from moderate to severe depression. The presence of any depression in this study was 25.4% (according to the PHQ-9), 5.8% participants had self-reported depression and 4.3% used anti-depressant medications.

**Table 1:** Baseline characteristics of participants (*N* = 397)

	Mean/ Numbers	SD/ Percentage
Age (years)	63.3	7.1
Female sex (n, %)	193	48.6
Body mass index (kg/m^2^)	29.6	4.9
Serum 25-(OH)D levels (nmol/L)	43.7	12.2
Treatment group (n, %)	201	50.6
***PHQ-9 score***		
0–4	296	74.6
5–9	70	17.6
≥ 10	31	7.8
Anti-depressant medication use (n, %)	17	4.3
***Education***		
School only (n, %)	49	12.4
High school (n, %)	96	24.4
University or higher (n, %)	249	63.2
***Work status***		
Full-time employed (n, %)	114	28.9
Part-time/causal employment (n, %)	83	21.0
Unemployed/home duties/retired (n, %)	198	50.1
***Work type***		
Manual (n, %)	128	32.8
Office/professional (n, %)	262	67.2
***WOMAC score***		
Pain (0–500)	136.4	87.0
Function (0–1700)	477.9	30.9
Stiffness (0–200)	62.0	40.7
***Multi-site joint pain, (n, %)***		
No pain	240	60.5
One site	133	33.5
More than one site	24	6.0
***Comorbidity, (n, %)***		
No comorbidity	162	40.8
One comorbidity	221	55.7
More than one comorbidity	14	3.5

*PHQ-9* Patient health questionnaire depression scale; *WOMAC* Western Ontario and McMaster Universities Arthritis Index

### Factors associated with the presence of depression

**Fig. 1:**
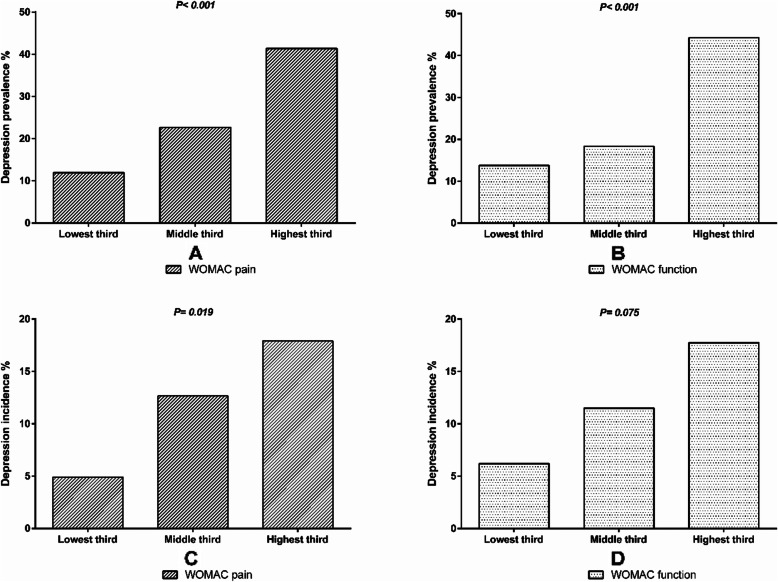
Associations of baseline WOMAC pain (0–500) and WOMAC function (0–1700) tertiles with the prevalence (**a**, **b**) and incidence (**c**, **d**) of depression in patients with knee OA

As shown in Table 2, being younger, female, and having a higher BMI, greater scores of WOMAC pain (Fig. 1a), WOMAC function (Fig. 1b) and WOMAC stiffness, a lower education level, and more than one comorbidity were significantly associated with a higher presence of depression in univariable analyses. After adjustment for age, sex, BMI and baseline 25-(OH)D level, the associations persisted except for female sex, which was no longer statistically significant. Having two or more sites of pain was significantly associated with a higher presence of depression in multivariable analyses. Age was not significantly associated with the presence of depression in either univariable or multivariable analysis. Results were similar, when self-reported depression was used as the outcome or if the analyses were adjusted for anti-depressant medication use (data not shown).

**Table 2:** Factors associated with the prevalence of mild to severe depression at baseline (*N* = 397)

	Univariable	Multivariable^a^
	PR (95% CI)	PR (95% CI)
Age (years)	**0.96 (0.94, 0.99)**	**0.96 (0.94, 0.99)**
Female sex (n, %)	**1.55 (1.10, 2.18)**	1.38 (0.98, 1.96)
BMI (kg/m^2^)	**1.05 (1.02, 1.09)**	**1.05 (1.02, 1.08)**
**Education**		
School only (n, %)	*Reference*	*Reference*
High school (n, %)	0.63 (0.40, 1.00)	**0.61 (0.39, 0.95)**
University or higher (n, %)	**0.49 (0.33, 0.73)**	**0.50 (0.34, 0.74)**
***Work status***		
Full-time employed (n, %)	*Reference*	*Reference*
Part-time/causal employment (n, %)	0.80 (0.49, 1.29)	0.78 (0.49, 1.25)
Unemployed/home duties/retired (n, %)	0.84 (0.57, 1.22)	1.10 (0.71, 1.70)
***Work type***		
Manual (n, %)	*Reference*	*Reference*
Office/professional (n, %)	0.88 (0.62, 1.25)	0.85 (0.60, 1.22)
**WOMAC score/ 10 unit**		
Pain (0–50)	**1.05 (1.03, 1.07)**	**1.05 (1.03, 1.07)**
Function (0–170)	**1.02 (1.01, 1.02)**	**1.02 (1.01, 1.02)**
Stiffness (0–20)	**1.08 (1.04, 1.12)**	**1.05 (1.02, 1.09)**
***Multi-site joint pain, (n, %)***		
No pain	*Reference*	*Reference*
One site	1.21 (0.85, 1.74)	1.06 (0.75, 1.51)
More than one site	1.64 (0.93, 2.88)	**1.73 (1.00, 2.98)**
***Comorbidity, (n, %)***		
No comorbidity	*Reference*	*Reference*
One comorbidity	1.08 (0.75, 1.55)	1.14 (0.80, 1.65)
More than one comorbidity	**2.13 (1.18, 3.86)**	**1.98 (1.03, 3.80)**

^a^ All multivariable analyses were adjusted for age, sex, BMI and baseline 25-(OH)D level, except for age (adjusted for sex, BMI and baseline 25-(OH)D level), sex (adjusted for age, BMI and baseline 25-(OH)D level) and BMI (adjusted for age, sex and baseline 25-(OH)D level)

Mild to severe depression was defined as PHQ-9 scores ≥5

### Factors associated with incident depression

Three hundred forty participants completed the follow-up. Twenty-eight out of 249 participants (11.2%) without depression at baseline had incident mild to severe depression at 24 months. Table 3 shows the factors associated with incident depression. Being female and having a higher WOMAC pain (Fig. 1c) and WOMAC function (Fig. 1d) score were significantly associated with greater incident depression over 24 months in univariable and multivariable analyses. The presence and incidence of depression were higher in females than in males (31.1% vs 20.1%, *p* = 0.01 for presence, and 27.3% vs 16.5%, *p* = 0.02 for incidence). Having two or more painful sites was significantly associated with greater incident depression over 24 months in the multivariable analyses. In a sensitivity analysis further adjusting for anti-depressant medication, the results were largely unchanged (data not shown). In contrast, there were no significant associations between age, BMI, education, WOMAC stiffness, comorbidity and incident depression.

**Table 3:** Factors associated with the incidence of mild to severe depression at 24 months amongst participants without depression at baseline (*N* = 249)

	Univariable	Multivariable^a^
	RR (95% CI)	RR (95% CI)
Age (years)	0.98 (0.94, 1.03)	1.00 (0.95, 1.04)
Female sex (n, %)	**2.47 (1.19, 5.13)**	**2.51 (1.17, 5.37)**
Body mass index (kg/m^2^)	1.00 (0.92, 1.09)	1.01 (0.93, 1.09)
**Education**		
School only (n, %)	*Reference*	*Reference*
High school (n, %)	0.82 (0.23, 2.88)	0.68 (0.20, 2.27)
University or higher (n, %)	0.75 (0.24, 2.34)	0.68 (0.23, 1.98)
***Work status***		
Full-time employed (n, %)	*Reference*	*Reference*
Part-time/causal employment (n, %)	0.85 (0.29, 2.55)	0.74 (0.25, 2.18)
Unemployed/home duties/retired (n, %)	1.22 (0.53, 2.81)	1.40 (0.54, 3.67)
***Work type***		
Manual (n, %)	*Reference*	*Reference*
Office/professional (n, %)	0.83 (0.40, 1.72)	0.68 (0.33, 1.38)
**WOMAC score/ 10 unit**		
Pain (0–50)	**1.05 (1.01, 1.09)**	**1.05 (1.02, 1.09)**
Function (0–170)	**1.01 (1.01, 1.03)**	**1.02 (1.01, 1.03)**
Stiffness (0–20)	1.03 (0.95, 1.12)	1.03 (0.95, 1.11)
***Multi-site joint pain, (n, %)***		
No pain	*Reference*	*Reference*
One site	1.43 (0.68, 3.01)	1.22 (0.58, 2.57)
More than one site	2.73 (0.91, 8.22)	**3.55 (1.31, 9.58)**
***Comorbidity, (n, %)***		
No comorbidity	*Reference*	*Reference*
One comorbidity	1.02 (0.50, 2.08)	1.13 (0.54, 2.38)
More than one comorbidity	1.51 (0.23, 9.83)	1.34 (0.21, 8.66)

^a^ All multivariable analyses were adjusted for age, sex, BMI, baseline 25-(OH)D level, and intervention, except for age (adjusted for sex, BMI baseline 25-(OH)D level and intervention), sex (adjusted for age, BMI, baseline 25-(OH)D level and intervention) and BMI (adjusted for age, sex, baseline 25-(OH)D level and intervention)

Mild to severe depression was defined as PHQ-9 scores ≥5

### Temporal relationship between baseline depression severity and changes in joint symptoms overtime

Table 4 describes the longitudinal association of baseline depression with changes in knee joint symptoms over 24 months. Although participants with mild to severe depression had greater decreases in WOMAC symptoms compared to participants without depression at baseline in the univariable analyses, the significant associations disappeared in the multivariable analyses. In addition, the results were consistent when either self-reported depression at baseline was used as the exposure or if the analyses were adjusted for anti-depressant medication use (data not shown). When considering the potential modification effect of treatment, the associations were examined separately for treatment and placebo groups. The results remained largely unchanged, except for the negative association between baseline depression and change in knee pain in the treatment group (Supplementary Table 1 and 2). Participants with mild or moderate/severe depression had significant decreases in WOMAC pain score in the treatment group, while participants with mild or moderate/severe depression had non-significant increases in WOMAC pain score in the placebo group. Similar trends were observed for change in WOMAC function and stiffness.

**Table 4:** The association between depression severity at baseline and change in joint symptom over 24 months (*N* = 337)

	Univariable β (95% CI)	Multivariable β (95% CI)
**WOMAC score**		
Pain (0–500) ^a^		
No depression	*Reference*	*Reference*
Mild depression	**−35.8 (−63.7, −7.9)**	−12.4 (−36.7, 11.8)
Moderate to severe depression	**−100.3 (− 143.1, −57.4)**	−32.0 (−72.2, 8.2)
Function (0–1700) ^b^		
No depression	*Reference*	*Reference*
Mild depression	**− 139.9 (− 222.2, − 57.7)**	−67.8 (− 145.4, 9.7)
Moderate to severe depression	**− 225.4 (− 357.5, −93.2)**	−90.3 (−220.3, 39.7)
Stiffness (0–200) ^c^		
No depression	*Reference*	*Reference*
Mild depression	**−13.2 (−26.0, −0.5)**	−5.2 (−16.5, 6.1)
Moderate to severe depression	**−29.4 (−49.0, − 9.8)**	−11.8 (−30.0, 6.5)

^a^ Multivariable analysis was adjusted for age, sex, BMI, baseline 25-(OH)D level, treatment arm and baseline WOMAC pain score

^b^ Multivariable analysis was adjusted for age, sex, BMI, baseline 25-(OH)D level, treatment arm and baseline WOMAC function score

^c^ Multivariable analysis was adjusted for age, sex, BMI, baseline 25-(OH)D level, treatment arm and baseline WOMAC stiffness score

## Discussion

This study investigated the temporal relationships between demographic and OA clinical factors, joint symptoms and depression in patients with symptomatic knee OA. Depression was common in this population with a presence of 25.4% and incidence of 11.2% over 24 months. Common OA risk factors such as higher BMI, lower education level and having two or more comorbidities were associated with prevalent depression and being female was associated with incident depression in knee OA patients, however, older age was negatively associated with prevalent depression. Higher levels of knee pain and physical dysfunction and having multi-site pain were associated with increased risks of both prevalent and incident depression. In contrast, baseline depression severity did not predict changes in OA symptoms. These findings provide epidemiological evidence that management of common OA risk factors, chronic pain and joint dysfunction may be beneficial for preventing and managing depression in knee OA patients.

Numerous studies have explored the demographic factors associated with depression in elderly populations. Female sex, lower education levels and the biological risks including endocrine and inflammatory factors are potential risk factors for depression in the elderly [24]. However, only a few studies have been conducted in individuals with OA, and most did not assess longitudinal relationships. A cross-sectional study reported that fewer social contacts, increased BMI, perceived pain and limited physical activity were associated with depression severity in 1021 patients with knee OA [15]. In our current study, we reported similar results and reported that, cross-sectionally, higher BMI, lower education level and having two or more comorbidities were associated with prevalent depression. Females had higher prevalent and incident depression than males, although the association of female sex with prevalent depression was not significant in multivariable analyses. While, we found younger age was associated with prevalent depression in elderly OA patients. Aging is a risk factor for OA, but not a risk factor for depression. It is noteworthy that the overall prevalence is lower in elderly compared to younger people, but older people with medical comorbidity have elevated risk for depression [25, 26].

Obesity, female sex and a lower education level are known as risk factors for both OA and depression. Therefore, the high prevalence depression in OA individuals may be in part attributed to shared risk factors. Taking higher BMI for example, higher BMI, indicating overweight or obesity, is a well-known risk factor of OA and also can increase vulenrability to depression directly and indirectly through complex mechanisms [27, 28]. Obesity causes a high prevalence of OA, which may be related to combined effects from biomechanical, inflammatory and metabolic factors. In addition, obesity can induce poor self-image, low self-esteem and social isolation, which are well-known contributors to depression. It also can activate inflammatory pathways, involving hypothamlamic-pituitary-adrenal axis dysreuglation, which are associated with increased risk of depression through biological and psychological pathways [27]. Obesity and lower levels of education are modifiable factors; therefore, management of obesity and improvement of education level may prevent depression and should have beneficial effects in patients with knee OA. Additionally, female sex predicted incident depression over 24 months in knee OA patients. Hence, it is important for clinicians to screen for, and try to prevent and treat depression, especially in female patients with knee OA.

Joint pain and dysfunction, multisite pain and comorbidities are clinical characteristics of OA. Multisite pain, joint pain and joint function limitation are common in musculoskeletal conditions, and have been linked to depression in previous studies [29]. Individuals who experienced chronic pain and physical activity limitation are at an increased risk of depression [30, 31]. In OA patients, joint pain severity and dsyfunction were associated with depression severity cross-sectionally and longitudinally [32–37]. Knee OA patients with greater pain associated with higher risk of depression at baseline and slow gait speed may represent an important risk factor for worsening depressive symptoms over time [35, 37]. The slow gait speed has been proved to be a risk factor for physical limitaion [38]. In our study, although we used a different method to assess depression severity, we found similar results. Individuals who experienced multi-site pain, more severe knee pain and joint dysfunction, and had more than one comorbidity were at a higher risk of prevalent depression cross-sectionally in knee OA patients. Over 24 months, multi-site pain, knee pain and dysfunction were associated with increased incidence of depression over 24 months, suggesting a potentially causal relationship. These supported the notion that management of pain, joint dysfunction and other comorbidities may help to improve depression in knee OA patients [17, 39].

Depression severity is dynamic, changing over time, and prior depressive illness modifies the experience of currently depressed mood [40]. The reciprocal relationship between depression severity and pain severity has been well established, but whether current depression severity has causal effects on severity of joint symptoms overtime has been rarely investigated [16, 18]. Kurt et al. have reported that change in pain severity over 3 months predicted subsequent depression severity, and vice versa, change in depression severity over 3 months predicted subsequent pain severity over 12 months in patients with persistent back, hip and knee pain [16]. The significant associations in the univariable analyses became non-significant after adjustment for co-variables, suggesting that the negative associations between baseline depression severity and change in WOMAC scores were confounded by other factors. In knee OA patient, we only found higher levels of knee joint symptoms and having multi-site pain at baseline were associated with increased risks of both prevalent and incident depression, while baseline depression severity did not predict knee joint symptomatic progression over 2 years. There was one study have explored the causal cumulative effect between depressive symptoms and knee pain among patients with knee radiographic OA. Rathbun reported the causal effect of pain severity significantly increases with the persistence of depressed mood, but depressive symptoms on OA knee pain does not change over time, which was smiliar with this current study [18]. Our findings suggest the potentially cause-effect relationship is from knee joint pain and physical dysfunction to depression, rather than from depression to knee symptoms, in patients with knee OA.

This study has some potential limitations. It was a post-hoc analysis of a randomized controlled trial which was primarily designed to examine vitamin D supplementation on knee OA outcomes. The social and socioeconomic deprivation, which are important factor and mediator for depression and the association with knee OA, had not been collected at enrollment, except for the educational status and employment information in this study. Therefore, this should be a limitation. Additionally, the generalisbility may be limited to OA patients recruited into trials, because of inherent issuses of secondary analysis of a RCT. Nevertheless, the findings from this study were plausible as we found that vitamin D supplementation reduced visual analog scale knee pain, improved physical function and decreased depessive symptoms [41, 42]. In this study, depression was associated with reduced knee pain and dysfunction over 2 years only in vitamin D supplemented group. The reasons underlying this are unclear but it may be related to effects of vitamin D treatment, as vitamin D supplementation reduced depessive symptoms as well as visual analog scale knee pain and dysfunction [41, 42]. Patients with depression would have a more responsiveness to vitamin D treatment and thus had further reductions in knee symptoms. In addition, 18% participants did not complete the 24 months follow-up, and loss of follow-up bias may be present; however, the retention rate in this trial was high, an there were no significant differences in the baseline characteristics between those who completed and who did not complete the trial. This suggests a minimal loss of follow-up bias in our study. Furthermore, we defined depression using the patient health questionnaire, which was developed for depression diagnostic, severity measures and assessment of depression outcome changes over time. It may lead to misclassification of depression; however, when we used the self-reported depression as the outcome or exposure and anti-depressant medication as a covariate and the results were largely unchanged.

## Conclusions

Knee OA risk factors and joint symptoms, along with co-existing multi-site pain are associated with the presence and development of depression. This suggests that managing common OA risk factors and joint symptoms may be important for prevention and treatment of depression in patients with knee OA.

## Supplementary Information

**Additional file 1.**

## Data Availability

The data that support the findings of this study are available from University of Tasmania Database, but restrictions apply to the availability of these data, which were used under license for the current study, and so are not publicly available. Data are available from the corresponding author upon reasonable request and with permission of University of Tasmania Database.

## Abbreviations

BMI: Body mass index
OA: Osteoarthritis
PHQ-9: Patient health questionnaire
SD: Standard deviation
VAS: Visual analogue scale
VIDEO: Vitamin D effect on osteoarthritis study
WOMAC: Western Ontario and McMaster universities osteoarthritis index
