# Supplementary material for: Depression in patients with knee osteoarthritis: risk factors and associations with joint symptoms
Source: BMC Musculoskelet Disord. 2021 Jan 7;22:40. doi: 10.1186/s12891-020-03875-1 (PMC7791830; doi:10.1186/s12891-020-03875-1)
Supplement: Supplementary file 1 — Additional file 1. [file 12891_2020_3875_MOESM1_ESM.docx]

*Supplementary Table 1. Factors associated with the incidence of mild to severe depression at 24 months amongst participants without depression* *at baseline*

|  | Treatment group  (N= 128) | Placebo group  (N= 121) | P-value for interaction |
| --- | --- | --- | --- |
|  | RR (95% CI) | RR (95% CI) |  |
| Age (years) | 0.98 (0.90, 1.06) | 1.01 (0.96, 1.07) | 0.526 |
| Female sex (n, %) | 2.16 (0.73, 6.38) | **3.06 (1.06, 8.83)** | 0.747 |
| Body mass index (kg/m^2^) | 1.00 (0.73, 6.38) | 1.02 (0.89, 1.16) | 0.775 |
| **Education** |  |  |  |
| School only (n, %) | *Reference* | *Reference* | *0.212* |
| High school (n, %) | 0.51 (0.15, 1.79) | 0.27 (0.05, 1.52) |  |
| University or higher (n, %) | 0.45 (0.17, 1.22) | 0.34 (0.10, 1.21) |  |
| ***Work status*** |  |  |  |
| Full-time employed (n, %) | *Reference* | *Reference* | *0.829* |
| Part-time/causal employment (n, %) | 1.05 (0.25, 4.45) | 0.50 (0.09, 2.71) |  |
| Unemployed/home duties/retired (n, %) | 2.24 (0.51, 9.78) | 0.99 (0.27, 3.68) |  |
| ***Work type*** |  |  |  |
| Manual (n, %) | *Reference* | *Reference* |  |
| Office/professional (n, %) | 1.40 (0.39, 4.96) | **0.34 (0.13, 0.89)** | 0.105 |
| **WOMAC score/ 10 unit** |  |  |  |
| Pain (0-50) | **1.05 (1.00, 1.10)** | **1.05 (1.00, 1.11)** | 0.852 |
| Function (0-170) | **1.02 (1.00, 1.03)** | **1.02 (1.00, 1.03)** | 0.954 |
| Stiffness (0-20) | 1.04 (0.95, 1.15) | 1.01 (0.89, 1.15) | 0.807 |
| ***Multi-site joint pain, (n, %)*** |  |  |  |
| No pain | *Reference* | *Reference* | *0.725* |
| One site | 1.39 (0.47, 4.13) | 1.10 (0.40, 3.00) |  |
| More than one site | **3.92 (1.17, 13.15)** | **3.56 (0.50, 25.38)** |  |
| ***Comorbidity, (n, %)*** |  |  |  |
| No comorbidity | *Reference* | *Reference* | *0.773* |
| One comorbidity | 0.94 (0.33, 2.65) | 1.30 (0.47, 3.63) |  |
| More than one comorbidity | 1.72 (0.22, 13.33) | 0.00 (0.00, 0.00) |  |

*All multivariable analyses were adjusted for age, sex, BMI,* *baseline 25-(OH)D level, and intervention, except for age (adjusted for sex, BMI and baseline 25-(OH)D level), sex (adjusted for age, BMI and baseline 25-(OH)D level) and BMI (adjusted for age, sex and baseline 25-(OH)D level).*

*Supplementary Table 2. The association between depression severity at baseline and change in joint symptom over 24 months*

|  | Treatment group  (N= 176)  β (95% CI) | Placebo group  (N= 161)  β (95% CI) | P-value for interaction |
| --- | --- | --- | --- |
| **WOMAC score** |  |  |  |
| Pain (0-500) † |  |  |  |
| No depression | *Reference* | *Reference* | *<0.001* |
| Mild depression | **-42.5 (-75.6, -9.5)** | 19.1 (-16.1, 54.4) |  |
| Moderate to severe depression | **-92.3 (-146.3, -38.4)** | 38.1 (-21.7, 98.9) |  |
| Function (0-1700) ‡ |  |  |  |
| No depression | *Reference* | *Reference* | *0.081* |
| Mild depression | -89.9 (-198.4, 18.7) | -27.2 (-149.4, 74.9) |  |
| Moderate to severe depression | -134.1 (-315.0, 46.8) | -54.4 (-245.3, 136.4) |  |
| Stiffness (0-200) § |  |  |  |
| No depression | *Reference* | *Reference* | *0.152* |
| Mild depression | -14.7 (-30.9, 1.5) | 6.5 (-9.3, 22.3) |  |
| Moderate to severe depression | -22.4 (-47.9, 3.0) | 1.3 (-25.2, 27.8) |  |

*† Multivariable analysis was adjusted for age, sex, BMI, baseline 25-(OH)D level and baseline pain score.*

*‡ Multivariable analysis was adjusted for age, sex, BMI, baseline 25-(OH)D level and baseline function score.*

*§ Multivariable analysis was adjusted for age, sex, BMI, baseline 25-(OH)D level and baseline stiffness score.*
